# Research on a Handheld 3D Laser Scanning System for Measuring Large-Sized Objects

**DOI:** 10.3390/s18103567

**Published:** 2018-10-21

**Authors:** Xiaomin Wang, Zexiao Xie, Kun Wang, Liqin Zhou

**Affiliations:** Engineering College, Ocean University of China, Qingdao 266100, China; wangkunouc@163.com (K.W.); liqin72@126.com (L.Z.)

**Keywords:** handheld 3D measurement system, binocular stereo vision, structured light, large-sized object, measurement on site

## Abstract

A handheld 3D laser scanning system is proposed for measuring large-sized objects on site. This system is mainly composed of two CCD cameras and a line laser projector, in which the two CCD cameras constitute a binocular stereo vision system to locate the scanner’s position in the fixed workpiece coordinate system online, meanwhile the left CCD camera and the laser line projector constitute a structured light system to get the laser lines modulated by the workpiece features. The marked points and laser line are both obtained in the coordinate system of the left camera in each moment. To get the workpiece outline, the handheld scanner’s position is evaluated online by matching up the marked points got by the binocular stereo vision system and those in the workpiece coordinate system measured by a TRITOP system beforehand; then the laser line with workpiece’s features got at this moment is transformed into the fixed workpiece coordinate system. Finally, the 3D information composed by the laser lines can be reconstructed in the workpiece coordinate system. A ball arm with two standard balls, which is placed on a glass plate with many marked points randomly stuck on, is measured to test the system accuracy. The distance errors between the two balls are within ±0.05 mm, the radius errors of the two balls are all within ±0.04 mm, the distance errors from the scatter points to the fitted sphere are distributed evenly, within ±0.25 mm, without accumulated errors. Measurement results of two typical workpieces show that the system can measure large-sized objects completely with acceptable accuracy and have the advantage of avoiding some deficiencies, such as sheltering and limited measuring range.

## 1. Introduction

Recently 3D contour measurement has been widely applied in many fields, such as heritage conservation, aerospace, automobile manufacturing and so forth. The coordinate measuring machine (CMM) and the articulated arm measurement system (AAMS) are the frequent-used 3D measurement devices. However, when measuring a large-sized object on site, the CMM is difficult to complete this task because it is not convenient being used on site. AAMS is portable and flexible, which could measure an object on site but its largest measurement scale is generally within several meters.

Now the 3D contour measurement based on optics [1,2,3,4,5,6] has been known as one of the most significant technologies with many advantages such as high accuracy, high efficiency, non-contact, which includes light coding, moire topography, structured light technique and space-time stereo vision and so forth. Light coding [7] is used to capture the movement features of human joints and figures but with low accuracy. Moire topography [8] could realize a measurement quickly but its capability is limited in estimating whether an object is concave or convex. Phase measurement profilometry (PMP) [9] can measure the contour of a moving object in real-time but it fails to find the optimum demodulation phase. Fourier transform frofilometry (FTP) [10] with high sensibility can obtain the surface points of an object only using one image captured with deformed grating fringes but its algorithm is very time-consuming. Handheld 3D line laser scanning [11] scans the contour online in several minutes by a structured light system composed by a fixed camera and handheld cross line laser projector, whose external parameters are obtained by self-calibration but its accuracy should be further improved. Spacetime stereo vision [12] can achieve the 3D contour measurement quickly by adding a projector to the binocular stereo vision system to solve the matching problem but its field-of-view is small. All these methods mentioned above measure an object from one view-point at a time. If the object needs to be measured completely, these methods have to measure the object from different view-points. Considering that each data patch has its own local coordinate system, data registration is necessary to acquire the complete surface points of the object in a common coordinate system with ineluctable registration errors at the junctions of different data patches. TRITOP system [13] has the ability to measure the rough geometry of a large-sized object on site by capturing pictures around the workpiece from different viewpoints and these pictures should include scale bars with known length. It is a non-contact measurement system proposed by Gom (Gesellschaft für Optische Messtechnik mbH) in Germany, with the primary ability of extracting the 3D coordinates of the marked points stuck on the objects. It has no limitation of measurement scale but it could not acquire the details of the 3D contour of objects. This study is developed to get the detail of the 3D contour of objects based on the TRITOP system.

In this paper, a handheld 3D laser scanning system is proposed to extract the 3D contours of large-sized objects on site. Its own measurement range is only several hundred millimeters. The extracted laser lines are within this range. In order to measure a large-sized object, the position and orientation of the scanning system is determined in real-time in the common workpiece coordinate system constructed by the known marked points randomly stuck on the workpiece. The positions of these marked points are measured by the TRITOP system beforehand. Then the laser points are transformed into the workpiece coordinate system continuously. Therefore, the system measuring range has no limitation just as TRITOP system and the measurement accuracy is largely dependent on the accuracy of TRITOP system.

## 2. System Composition and Working Principle

**Figure 1:**
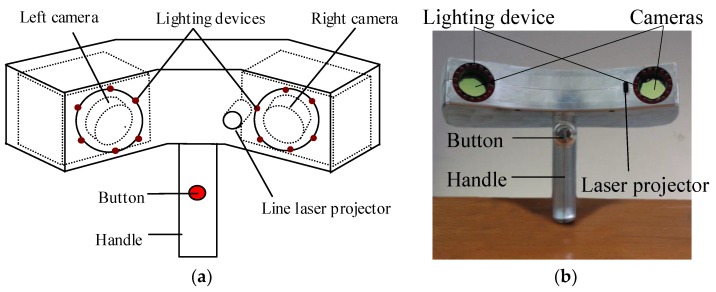
System composition and structure (**a**) schematic diagram; (**b**) picture of measurement system.

As shown in Figure 1, this measurement system is mainly composed of two CCD cameras, a line laser projector and two sets of lighting devices. The two CCD cameras constitute a binocular stereo vision system, while the left CCD camera and the line laser projector constitute a structured light system. The coordinate system of left camera is defined as the sensor coordinate system, noted as the $o_{1}x_{1}y_{1}z_{1}$.

**Figure 2:**
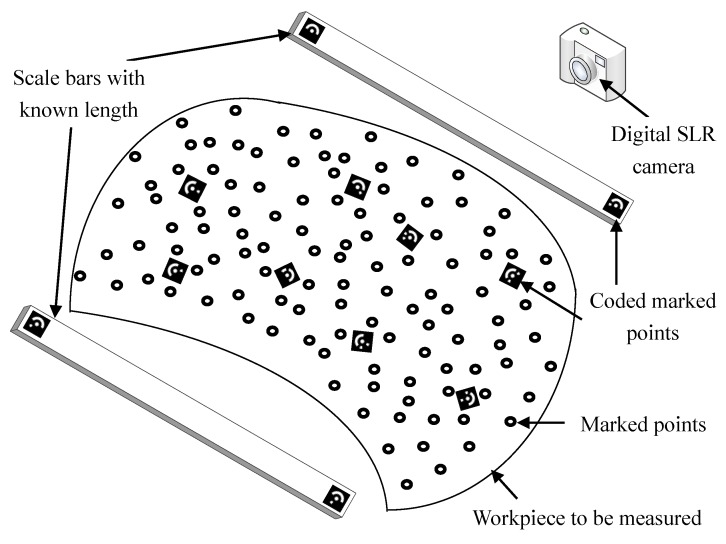
Working principle of TRITOP.

At the beginning, the marked points are randomly stuck on the surface of one workpiece and then measured by TRITOP system. The working principle of TRITOP system is shown in Figure 2 [14]. Two scale bars are put aside of the workpiece to be measured, the scale bars lengths between the coded marked points at both ends are known. Some coded marked points are put on the workpiece for the orientation of 2D images. In the next step, one digital SLR camera with fixed focal length of 24 mm is used to take pictures around the workpiece from different viewpoints and each picture should include at least one scale bar in its entirety and at least 5 coded marked points. Then these pictures and the parameters of the digital camera are input to its own software, “TRITOP Professional”. Finally, the 3D coordinates of the marked points are obtained with all the relative distance errors within 0.2 mm and a 3D coordinate system is established on the fixed workpiece, defined as workpiece coordinate system $o_{w}x_{w}y_{w}z_{w}$.

**Figure 3:**
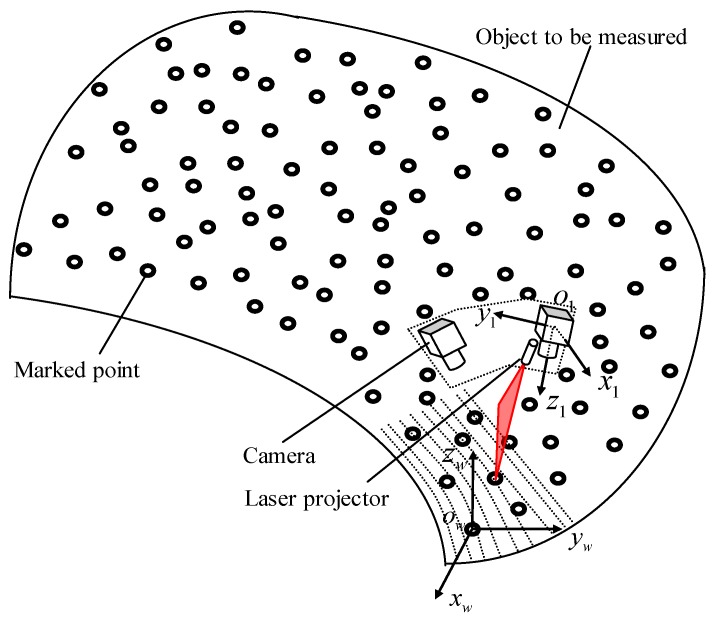
System working principle.

**Figure 4:**
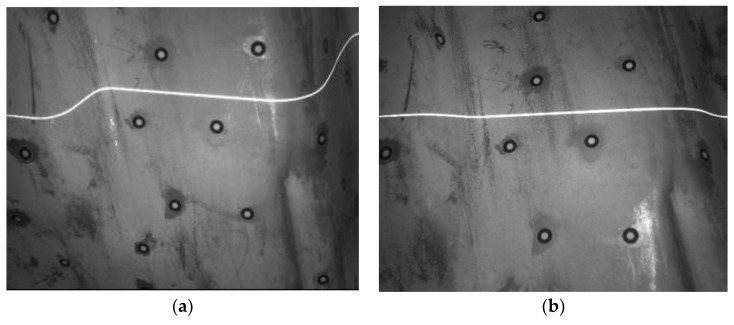
Two images containing marked points and a laser stripe captured with small aperture to get clear laser stripe. (**a**) Left image; (**b**) right image.

With the 3D coordinates of marked points in $o_{w}x_{w}y_{w}z_{w}$, the working principle of this study to scan the 3D contour of workpiece is shown in Figure 3. At each position, the system captures two images, shown in Figure 4, each of them contains several marked points and a laser stripe modulated by the workpiece features. Then the 3D coordinates of the marked points and the laser stripe are calculated in $o_{1}x_{1}y_{1}z_{1}$ according to the binocular stereo vision model and the structured light model respectively. To achieve 3D contour of the entire workpiece, the laser stripe in $o_{1}x_{1}y_{1}z_{1}$ should be transformed into $o_{w}x_{w}y_{w}z_{w}$. The transformation is solved by using the 3D coordinates of the detected marked points in $o_{1}x_{1}y_{1}z_{1}$ and their corresponding coordinates in $o_{w}x_{w}y_{w}z_{w}$ measured by TRITOP system. After scanning over the workpiece, the laser points in $o_{w}x_{w}y_{w}z_{w}$ can make up the contour of workpiece.

Therefore, to achieve the 3D contour of large-sized workpiece accurately, the binocular stereo vision model and the structured light model should be modeled and calibrated; the corresponding coordinates in $o_{w}x_{w}y_{w}z_{w}$ of the marked points in $o_{1}x_{1}y_{1}z_{1}$ should be found out to compute the transformation relationship between current $o_{1}x_{1}y_{1}z_{1}$ and $o_{w}x_{w}y_{w}z_{w}$.

The organization of this paper is as follows: Section 3 presents the modeling and calibration of the binocular stereo vision system and the structured light system, in which the 3D coordinates of the marked points and laser points are achieved in $o_{1}x_{1}y_{1}z_{1}$. Section 4 addresses the coordinate match-up method of the same marked points in $o_{1}x_{1}y_{1}z_{1}$ and in $o_{w}x_{w}y_{w}z_{w}$. Using the matched-up coordinates, the transformation from $o_{1}x_{1}y_{1}z_{1}$ into $o_{w}x_{w}y_{w}z_{w}$ is achieved. The 3D laser data obtained in $o_{1}x_{1}y_{1}z_{1}$ can then be transformed into $o_{w}x_{w}y_{w}z_{w}$. Section 5 describes the experiments and gives out the accuracy analysis. The conclusion is given in Section 6.

## 3. Modeling and Calibration of the Handheld 3D Laser Scanning System

In order to achieve the 3D contours of given large-sized objects accurately, the internal and external parameters of both the binocular stereo vision system and structured light system should be modeled and calibrated.

### 3.1. Modeling and Calibration of the Binocular Stereo Vision System

**Figure 5:**
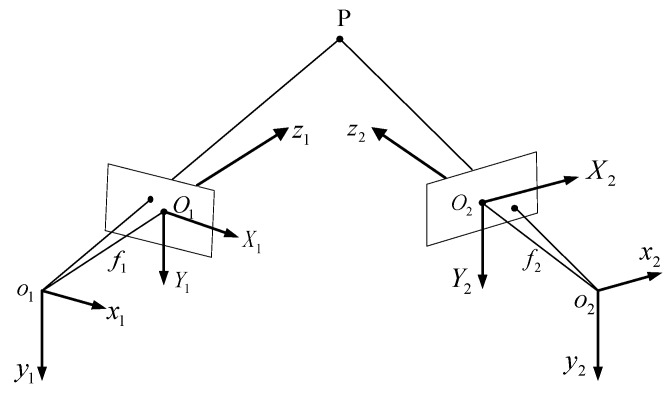
Binocular stereo vision system model.

Shown in Figure 5, $o_{1}x_{1}y_{1}z_{1}$ is the left camera coordinate system, also defined as the scanner coordinate system. According to the perspective projection principle, the transformation from the camera coordinate system to the CCD array plane is shown in Equations (1) and (2), respectively.

(1) $$\rho_{1}\left[\begin{array}{c} X_{1}\\ Y_{1}\\ 1 \end{array}\right]=\left[\begin{array}{ccc} f_{1} & 0 & 0\\ 0 & f_{1} & 0\\ 0 & 0 & 1 \end{array}\right]\left[\begin{array}{c} x_{1}\\ y_{1}\\ z_{1} \end{array}\right]\;$$

(2) $$\rho_{2}\left[\begin{array}{c} X_{2}\\ Y_{2}\\ 1 \end{array}\right]=\left[\begin{array}{ccc} f_{2} & 0 & 0\\ 0 & f_{2} & 0\\ 0 & 0 & 1 \end{array}\right]\left[\begin{array}{c} x_{2}\\ y_{2}\\ z_{2} \end{array}\right]\;$$

where $\rho_{1}$ and $\rho_{2}$ are scale factors.

Only taking radial distortion into consideration, the relationship between the distorted position $P_{id}(X_{id},Y_{id})$ and ideal position $P(X_{i},Y_{i})$ in $O_{i}X_{i}Y_{i}$ is

(3) $$\{\begin{array}{c} X_{i}=X_{id}(1+k_{i1}q^{2}+k_{i2}q^{4})\\ Y_{i}=Y_{id}(1+k_{i1}q^{2}+k_{i2}q^{4}) \end{array}\;$$

where i=1,2 represents two cameras, $q^{2}=X_{id}{}^{2}+Y_{id}{}^{2}={\left(\frac{u_{d}-u_{i0}}{N_{x}}\right)}^{2}+{\left(\frac{v_{d}-v_{i0}}{N_{y}}\right)}^{2}$, $\left(u_{i0},v_{i0}\right)$ are the principal point of both cameras, $k_{i1}$ and $k_{i2}$ are the first-order and second-order distortion coefficients of both cameras.

The transformation from $o_{1}x_{1}y_{1}z_{1}$ to $o_{2}x_{2}y_{2}z_{2}$ is

(4) $$P_{2}=M_{1}P_{1}=\left[\begin{array}{cc} R_{1} & T_{1} \end{array}\right]P_{1},\;R_{1}=\left[\begin{array}{ccc} r_{11} & r_{12} & r_{13}\\ r_{14} & r_{15} & r_{16}\\ r_{17} & r_{18} & r_{19} \end{array}\right],T_{1}=\left[\begin{array}{c} t_{1x}\\ t_{1y}\\ t_{1z} \end{array}\right].$$

where $P_{1}={\left[\begin{array}{ccc} x_{1} & y_{1} & z_{1} \end{array}\right]}^{T}$ and $P_{2}={\left[\begin{array}{ccc} x_{2} & y_{2} & z_{2} \end{array}\right]}^{T}$ are the coordinates of one 3D point in $o_{1}x_{1}y_{1}z_{1}$ and $o_{2}x_{2}y_{2}z_{2}$ respectively, $R_{1}$ is a 3 × 3 rotation matrix from $o_{1}x_{1}y_{1}z_{1}$ to $o_{2}x_{2}y_{2}z_{2}$, $T_{1}$ is a translation vector.

From Equations (1), (2) and (4), the transformation from $O_{1}X_{1}Y_{1}$ to $O_{2}X_{2}Y_{2}$ is derived as

(5) $$\rho_{2}\left[\begin{array}{c} X_{2}\\ Y_{2}\\ 1 \end{array}\right]=\left[\begin{array}{cccc} f_{2}r_{11} & f_{2}r_{12} & f_{2}r_{13} & f_{2}t_{1x}\\ f_{2}r_{14} & f_{2}r_{15} & f_{2}r_{16} & f_{2}t_{1y}\\ r_{17} & r_{18} & r_{19} & t_{1z} \end{array}\right]\left[\begin{array}{c} z_{1}X_{1}/f_{1}\\ z_{1}Y_{1}/f_{1}\\ z_{1}\\ 1 \end{array}\right]\;$$

Then the 3D coordinate of one point in $o_{1}x_{1}y_{1}z_{1}$ can be calculated from Equation (6), after the unknown internal and external parameters in Equations (1)–(4) are calibrated.

(6) $$\left\{ \begin{array}{l} x_{1}=z_{1}X_{1}/f\\ y_{1}=z_{1}Y_{1}/f\\ z_{1}=\frac{f_{1}(f_{2}t_{1y}-Y_{2}t_{1z})}{Y_{2}(r_{17}X_{1}+r_{18}Y_{1}+f_{1}r_{19})-f_{2}(r_{14}X_{1}+r_{15}Y_{1}+f_{1}r_{16})} \end{array}\right.\;$$

**Figure 6:**
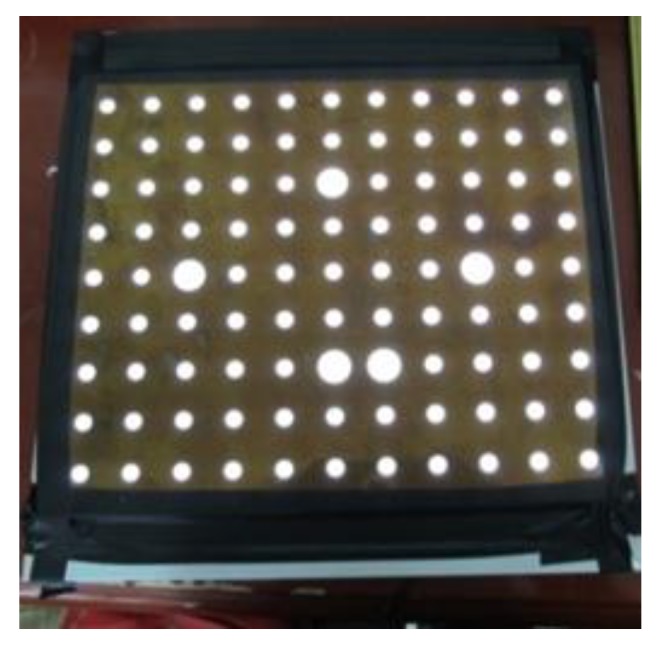
Target for binocular vision calibration.

The binocular stereo vision system is calibrated by adopting the calibration target shown in Figure 6. To make the calibration results accurate, the target points should fill the whole view field and the postures of target in the stereo system should be fully considered. As a result, five poses set calibration method is introduced to get the calibration points [15,16]. The calibration points are extracted from15 pair images with 5 different postures and 3 pair images captured at each posture.

The 2D coordinates of the marked points are extracted from these 15 pairs of images with sub-pixel precision [17]. The unknown parameters in Equations (1)–(4), including $R_{1}$, $T_{1}$, $f_{1}$, $f_{2}$, $u_{10}$, $v_{10}$, $u_{20}$, $v_{20}$, $k_{11}$, $k_{12}$, $k_{21}$ and $k_{22}$, are then calibrated by adopting the binocular stereo calibration function of OpenCV.

### 3.2. Modeling and Calibration of the Structured Light System

**Figure 7:**
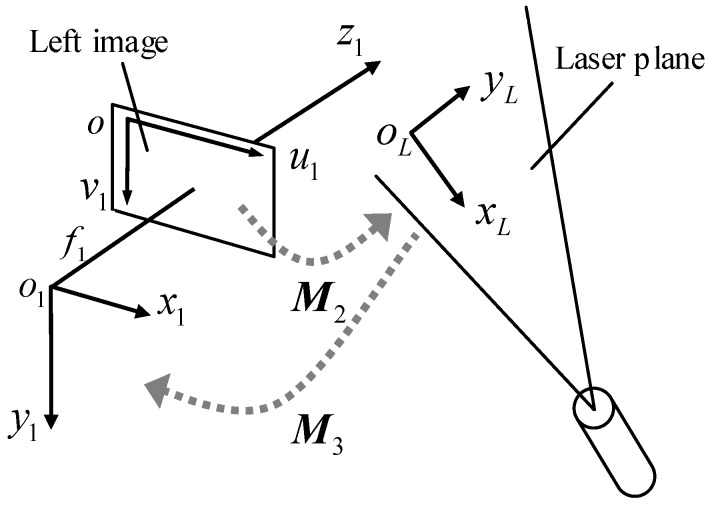
Structured light system model.

The principle of the structured light system is based on the triangulation method shown in Figure 7. The relative position between the left image and the laser plane is optimally designed for achieving a satisfying working depth and measurement accuracy. The model of the structured light system should be the transformation from the 2D image plane to the 2D laser plane where a 2D coordinate system $o_{L}x_{L}y_{L}$ is established, it is a one-to-one mapping relationship and can be created as

(7) $$\rho_{3}\tilde{P}=M_{2}{\tilde{P}}_{L},\;M_{2}=\left[\begin{array}{ccc} a_{1} & a_{2} & a_{3}\\ a_{4} & a_{5} & a_{6}\\ a_{7} & a_{8} & 1 \end{array}\right]\;$$

where $\tilde{P}={\left[\begin{array}{ccc} u_{1} & v_{1} & 1 \end{array}\right]}^{T}$ and ${\tilde{P}}_{L}={\left[\begin{array}{ccc} x_{L} & y_{L} & 1 \end{array}\right]}^{T}$ are the homogeneous coordinates of one calibration point in $ou_{1}v_{1}$ and in $o_{L}x_{L}y_{L}$ respectively, $a_{1}\sim a_{8}$ are defined as intrinsic parameters.

In Equation (7), $\left(u_{1},v_{1}\right)$ is a coordinate on the image plane after the lens distortions are corrected using the calibrated distortion coefficients in Section 3.1. Once the intrinsic parameters $a_{1}\sim a_{8}$ are calibrated, the 2D coordinate of a point $\left(x_{L},y_{L}\right)$ on a laser stripe can be obtained from the $\left(u_{1},v_{1}\right)$ on the image plane.

To achieve 3D measurement, it is necessary to transform the 2D laser points in $o_{L}x_{L}y_{L}$ into $o_{1}x_{1}y_{1}z_{1}$, such a transformation is the extrinsic model of the structured light system and it is created as

(8) $$P_{1}=M_{3}{\tilde{P}}_{L}=\left[\begin{array}{cc} R_{3} & T_{3} \end{array}\right]{\tilde{P}}_{L},\;R_{3}=\left[\begin{array}{cc} r_{31} & r_{34}\\ r_{32} & r_{35}\\ r_{33} & r_{36} \end{array}\right],T_{3}=\left[\begin{array}{c} t_{3x}\\ t_{3y}\\ t_{3z} \end{array}\right].$$

where $P_{1}={\left[\begin{array}{ccc} x_{1} & y_{1} & z_{1} \end{array}\right]}^{T}$ is the coordinate of a 3D calibration point in $o_{1}x_{1}y_{1}z_{1}$. ${\tilde{P}}_{L}={\left[\begin{array}{ccc} x_{L} & y_{L} & 1 \end{array}\right]}^{T}$ is the homogeneous coordinate of a 2D calibration point in $o_{L}x_{L}y_{L}$. $R_{3}$ is a 3 × 2 rotation matrix, which includes the unit direction vectors of $x_{L}$ and $y_{L}$ axes in $o_{1}x_{1}y_{1}z_{1}$ and $T_{3}$ is the position of $o_{L}$ in $o_{1}x_{1}y_{1}z_{1}$. They are the extrinsic parameters to be calibrated.

For solving the intrinsic and extrinsic parameters, the calibration points in the laser plane need to be established. During this process, the LED light devices are not working to extract accurately the laser points. Firstly, the laser plane is projected on a glass plate painted with white matt paint. The laser stripe on the plate is captured by both cameras. Secondly, the center position of the laser stripe in $ou_{1}v_{1}$ is extracted using the gray-weight centroid method [18,19,20]. Thirdly, a point on the laser stripe in the left image is matched up with its corresponding point in the right image according to the epipolar geometry constraint. The 3D coordinate of the laser points in $o_{1}x_{1}y_{1}z_{1}$ is calculated from Equation (6).

**Figure 8:**
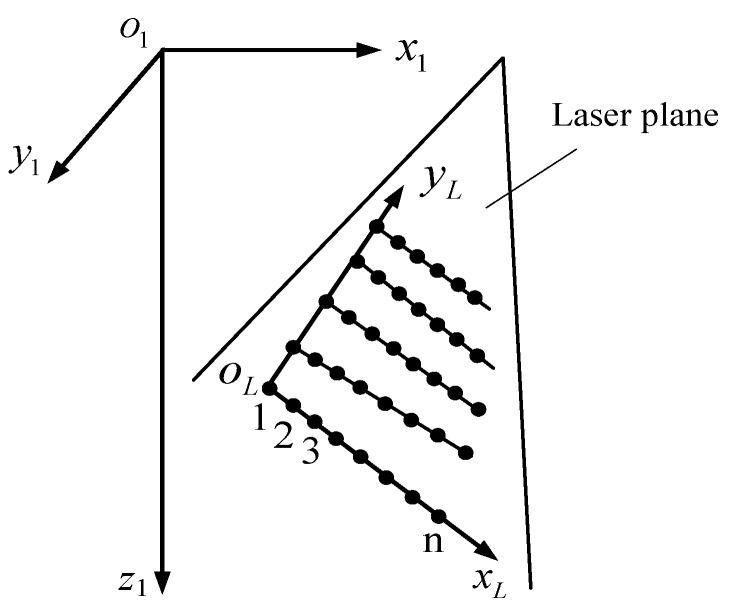
Relationship between $o_{1}x_{1}y_{1}z_{1}$ and $o_{L}x_{L}y_{L}$.

Following the process described above, several laser stripes are obtained at different positions to keep the laser points distributed in different regions of the laser plane, shown in Figure 8. Since the laser stripe projected on the glass plate is a line, the 3D points on it are co-linear in $o_{1}x_{1}y_{1}z_{1}$. In Figure 8, 1,2,3…,n are co-linear points. They are applied to fit a line, direction of the line is from 1 to *n* which can be regarded as the direction of an axis in the laser plane, defined as $x_{L}$, its direction vector in $o_{1}x_{1}y_{1}z_{1}$ is ${\left[\begin{array}{ccc} r_{31} & r_{32} & r_{33} \end{array}\right]}^{T}$. Subsequently, all of the collected points in $o_{1}x_{1}y_{1}z_{1}$ are used to fit a plane, the normal direction of the plane is computed as n. From ${\left[\begin{array}{ccc} r_{31} & r_{32} & r_{33} \end{array}\right]}^{T}$ and n, an axis perpendicular to ${\left[\begin{array}{ccc} r_{31} & r_{32} & r_{33} \end{array}\right]}^{T}$ in the laser plane can be determined, defined as $y_{L}$ and its direction vector is ${\left[\begin{array}{ccc} r_{34} & r_{35} & r_{36} \end{array}\right]}^{T}$, according to the right hand rule. If $y_{L}$ passes through point 1, the origin of the 2D coordinate frame is located at point 1 whose coordinate is ${\left(t_{3x},t_{3y},t_{3z}\right)}^{T}$.

As a result, a 2D coordinate frame $o_{L}x_{L}y_{L}$ is established in the laser plane, the coordinate of $o_{L}$ and the direction vectors of $x_{L}$ and $y_{L}$ in $o_{1}x_{1}y_{1}z_{1}$ are simultaneously solved while establishing $o_{L}x_{L}y_{L}$. The extrinsic parameters in Equation (8) is then solved. To calibrate the intrinsic parameters, the 2D calibration points in the laser plane should be established by transforming the 3D calibration points in $o_{1}x_{1}y_{1}z_{1}$ into $o_{L}x_{L}y_{L}$. Deriving from Equation (8), we have

(9) $${\tilde{P}}_{L}={\left(M_{3}\right)}^{-1}P_{1}$$

With all the calibration points in $o_{L}x_{L}y_{L}$ and their corresponding points in $ou_{1}v_{1}$, the intrinsic parameters $a_{1}\sim a_{8}$ in Equation (7) are worked out.

## 4. Match-Up Method of the Marked Points in $o_{1}x_{1}y_{1}z_{1}$ and $o_{w}x_{w}y_{w}z_{w}$

### 4.1. Matching Up the Marked Points

The objective of matching up the marked points is to identify the coordinates of the same marked point in $o_{1}x_{1}y_{1}z_{1}$ and in $o_{w}x_{w}y_{w}z_{w}$. In this study, this is achieved by using a distance constraint algorithm.

The position of each marked point in $o_{w}x_{w}y_{w}z_{w}$ obtained by TRITOP has a sequence number. To match up the marked points in both $o_{1}x_{1}y_{1}z_{1}$ and $o_{w}x_{w}y_{w}z_{w}$, the known marked points in $o_{w}x_{w}y_{w}z_{w}$ should be assigned according to relative distances between marked points.

Firstly, the distances $d_{ij}$ between any two marked points are calculated in $o_{w}x_{w}y_{w}z_{w}$. Considering the small working range of binocular stereo vision system, the maximum distance between two marked points will be within $d_{\max}\leq 300\;\mathrm{mm}$ in $o_{1}x_{1}y_{1}z_{1}$. Then all of distances between marked points in $o_{w}x_{w}y_{w}z_{w}$ meeting this condition, $d_{ij}\leq 300\;\mathrm{mm}$, will be sorted according to the ascending order and stored in a library (noted as $L_{w}=\{P_{i},d_{ij},P_{j}\}$, j≠i) and each distance has a corresponding node. The library is shown below, 
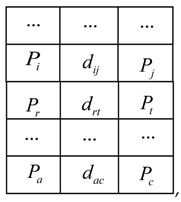
 where $P_{i}$, $P_{j}$, $P_{r}$, $P_{t}$, $P_{a}$, $P_{c}$ are marked points with the sequence number *i*, *j*, *r*, *t*, *a*, *c* in $o_{w}x_{w}y_{w}z_{w}$ respectively, $d_{ij}$, $d_{rt}$, $d_{ac}$ are the distance between them respectively and $\ldots \leq d_{ij}\leq d_{rt}\leq \ldots d_{ac}$.

Then for each marked point $P_{i}$, a workpiece sub-library (noted as $L_{wi}$, i={1,2,…,n}, n is the number of marked points) is constructed by $P_{i}$ and its neighbors (the points connected with $P_{i}$ meeting $d_{ij}\leq 300\;\mathrm{mm}$), which is also sorted ascendingly according to the distances. One workpiece sub-library, $L_{wi}$, is 
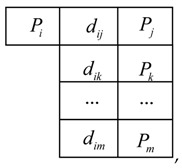
 where points $P_{i}$ are connected with points $P_{j}$, $P_{k}$, …, $P_{m}$ and $d_{ij}\leq d_{ik}\leq \ldots \leq d_{im}\leq 300\;\mathrm{mm}$.

**Figure 9:**
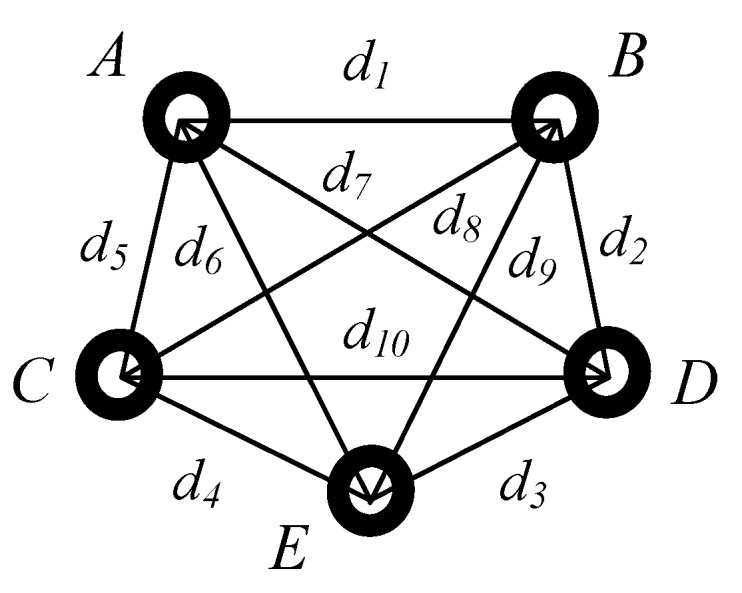
The web $W_{t_{j}}$ constituted by the marked points obtained in $o_{1}x_{1}y_{1}z_{1}$ at the moment $t_{j}$.

**Figure 10:**
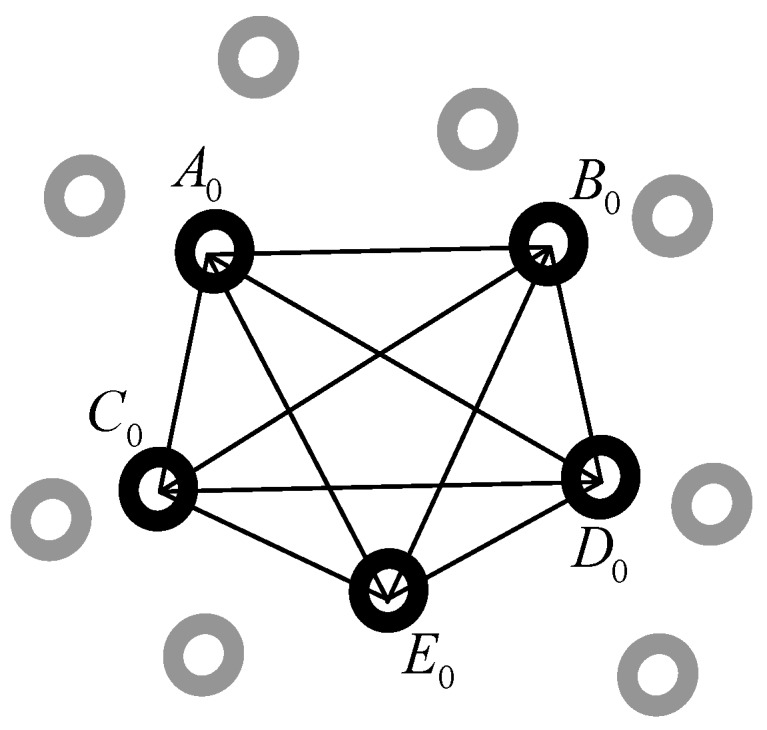
Corresponding marked points to Figure 9 and the web $W_{i}$ in $o_{w}x_{w}y_{w}z_{w}$. The black marked points are the same points with the points in Figure 9, while the gray marked points are other points stuck on the workpiece.

Then the handheld scanner begins scanning the workpiece’s contour. For example, $n_{t_{j}}$ marked points are obtained in $o_{1}x_{1}y_{1}z_{1}$ at the moment $t_{j}$ and the distances between arbitrary two marked points are calculated. Similar to $L_{wi}$, the scanner sub-libraries (noted as $L_{sj}$, $j=\{1,2,\ldots ,n_{t_{j}}\}$) are created for each of these marked points and these marked points make up of a web, noted as $W_{t_{j}}$ (see Figure 9). As all of the marked points are fixed on the workpiece, there must exist a same web in $o_{w}x_{w}y_{w}z_{w}$, shown in Figure 10. Since the marked points are randomly stuck on the workpiece, there is one and only one web $W_{i}$ in $o_{w}x_{w}y_{w}z_{w}$ as same as the web $W_{t_{j}}$ in Figure 9.

To find the web $W_{i}$ in $o_{w}x_{w}y_{w}z_{w}$, a distance constraint algorithm employed is explained below (the web in Figure 9 is as an example):

(1) Choose one marked point, such as $P_{A}$, in Figure 9 with its scanner sub-library $L_{sA}$ in $o_{1}x_{1}y_{1}z_{1}$;
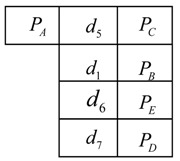
(2) find the distances in library $L_{w}$ meeting the conditions $\left|d_{5}-d_{ij}\right|\leq \varepsilon$ and record the sequences of the marked points to a list. Assuming $\left|d_{5}-d_{ij}\right|\leq \varepsilon$ and $\left|d_{5}-d_{rt}\right|\leq \varepsilon$, then the list of candidates is generated, $l_{c}=\{i,j,r,t\}$.
(3) check the workpiece sub-libraries according to the list $l_{c}$,$\left\{L_{wi},L_{wj},L_{wr},L_{wt}\right\}$, successively to find whether it exists all the distances $d_{*m}$ meeting the conditions $\left|d_{k}-d_{*m}\right|\leq \varepsilon$(k=1,6,7, ∗=i,j,r,t and m stands for one distance in workpiece sub-libraries $L_{w*}$) respectively. For instance, if the distances $d_{jm}$ in $L_{wj}$ meet to $\left|d_{k}-d_{*m}\right|\leq \varepsilon$, then the point $P_{j}$ is believed as the same point with $P_{A}$ in $o_{w}x_{w}y_{w}z_{w}$.
(4) repeat the steps (1)~(3) to find the points corresponding to $P_{B}$, $P_{C}$, $P_{D}$, $P_{E}$, then the marked points are matched up well.

### 4.2. Obtaining the Contour of Workpiece in $o_{w}x_{w}y_{w}z_{w}$

To get the whole contour of one workpiece, the laser lines should be transformed into $o_{w}x_{w}y_{w}z_{w}$ from $o_{1}x_{1}y_{1}z_{1}$. To do this, the transformation relationship between these two frames (see Equation (10)) should be worked out firstly by the matched-up marked points in Section 4.1.

(10) $$P_{w}=M_{4}{\tilde{P}}_{1}=\left[\begin{array}{cc} R_{4} & T_{4} \end{array}\right]{\tilde{P}}_{1},\;R_{4}=\left[\begin{array}{ccc} r_{41} & r_{42} & r_{43}\\ r_{44} & r_{45} & r_{46}\\ r_{47} & r_{48} & r_{49} \end{array}\right],T_{4}=\left[\begin{array}{c} t_{4x}\\ t_{4y}\\ t_{4z} \end{array}\right]\;$$

where $R_{4}$ is a 3 × 3 rotation matrix from $o_{1}x_{1}y_{1}z_{1}$ to $o_{w}x_{w}y_{w}z_{w}$, $T_{4}$ is a translation vector. $P_{w}={\left[\begin{array}{ccc} x_{w} & y_{w} & z_{w} \end{array}\right]}^{T}$ and $P_{1}={\left[\begin{array}{ccc} x_{1} & y_{1} & z_{1} \end{array}\right]}^{T}$ are the 3D coordinates of a same 3D point in $o_{w}x_{w}y_{w}z_{w}$ and $o_{1}x_{1}y_{1}z_{1}$, respectively.

Then the laser lines got from the structured light system can be transformed into the fixed $o_{w}x_{w}y_{w}z_{w}$. When the handheld scanner finishes the scanning process, all the laser lines modulated by the workpiece’s features are transformed into $o_{w}x_{w}y_{w}z_{w}$. The achievable result will be got.

## 5. Experiments and Results

### 5.1. System Hardware and Structure

**Figure 11:**
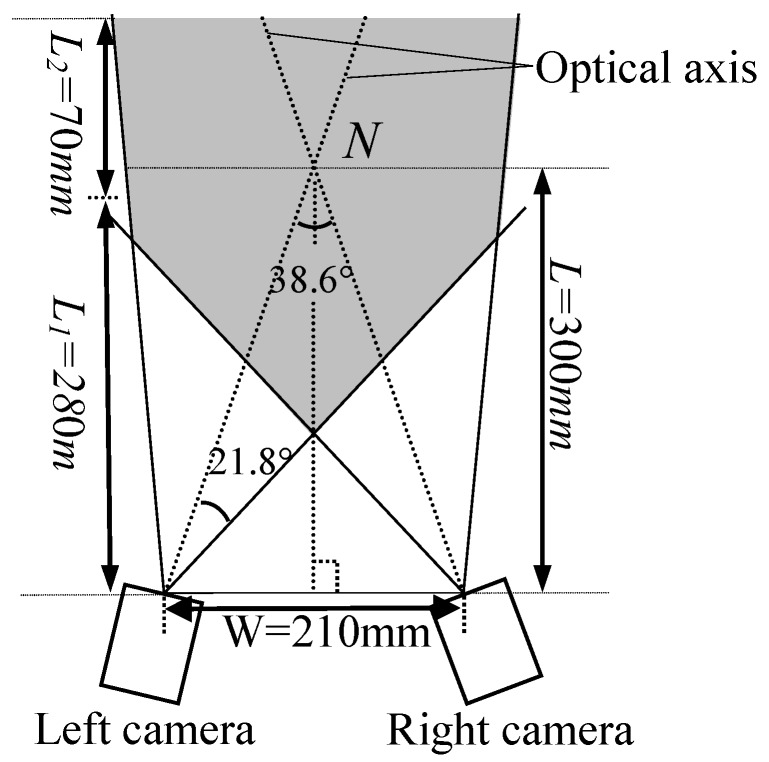
Field-of-view of the measurement system. The distance L from the intersection point of two optical axes N to the line between the optical points of the two cameras is designed as L=300 mm and the angle of the two optical axes is designed as 38.6°. The depth of field should be kept within a suitable range ($280\;\mathrm{mm}\leq L_{2}\leq 350\;\mathrm{mm}$ in this study). The gray range is the effective view field of the system.

The system is shown in Figure 1b. Two cameras are made by Pointgrey, Canada, with the model FL3-FW-03S3M. The resolution of the CCD array plane is 640 × 480. The frame rate of the two cameras is set as 60 frames/s, the shutter time as 8 ms, the gain as 6 dB. The lenses are generated by Computar, with 8 mm fixed focal length for $1/2^{\prime \prime }$ format sensors. To obtain clear laser lines, a small aperture is adopted. Moreover, to achieve an appropriate measuring range, the distance L from the intersection point of two optical axes N to the line between the optical points of the two cameras is designed as L=300 mm and the angle of the two optical axes is designed as 38.6° considering two factors: (1) at least 5 same marked points can be synchronously captured to a large extent by the two cameras for the binocular stereo matching up; (2) the depth of field should be kept within a suitable range ($280\;\mathrm{mm}\leq L_{2}\leq 350\;\mathrm{mm}$ in this study) for requiring a satisfying accuracy. As shown in Figure 11, the gray area is the effective view field.

### 5.2. System Accuracy Test

**Figure 12:**
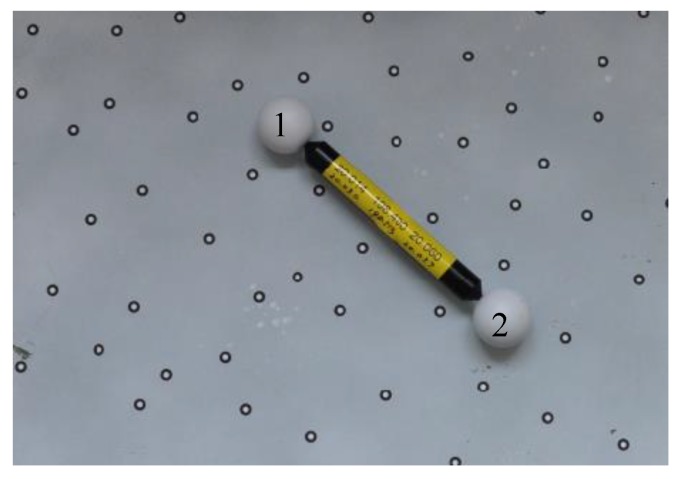
The device used to test the accuracy of the system. The glass plate (400 mm×500 mm) is painted with white matt paint, in which 69 marked points are struck on with known 3D coordinates in $o_{w}x_{w}y_{w}z_{w}$ measured accurately by TRITOP system. A ball arm with two standard spheres is put on this glass plate to test the accuracy of the system, the standard radii of sphere 1 and sphere 2 and their distance are 20.030 mm, 20.057 mm, 198.513 mm, respectively.

In order to test the accuracy of the system, the device in Figure 12 is adopted, which includes a glass plate painted with white matt paint and a ball arm with two standard spheres. The size of the glass plate is 400 mm × 500 mm, in which 69 marked points were randomly stuck on the glass plane and their 3D coordinates in the $o_{w}x_{w}y_{w}z_{w}$ were measured accurately using TRITOP system beforehand. The radius of sphere 1 is 20.030 mm, the radius of sphere 2 is 20.057 mm. The distance between them is 198.513 mm.

As the ball arm is placed on the glass plate motionlessly, they can be regarded as one object. The system can simultaneously measure the glass plate and the ball arm. As a result, the systematic error and random error of the system can be estimated by using the obtained surface points on the two spheres.

#### 5.2.1. Systematic Error Test

**Figure 13:**
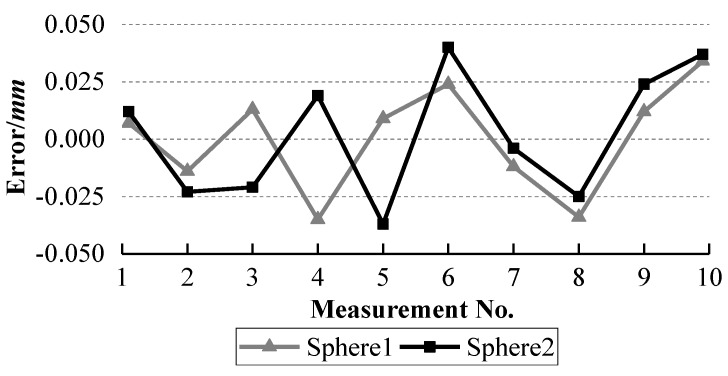
Errors between the standard radii and the measured radii of the two spheres with our system.

**Figure 14:**
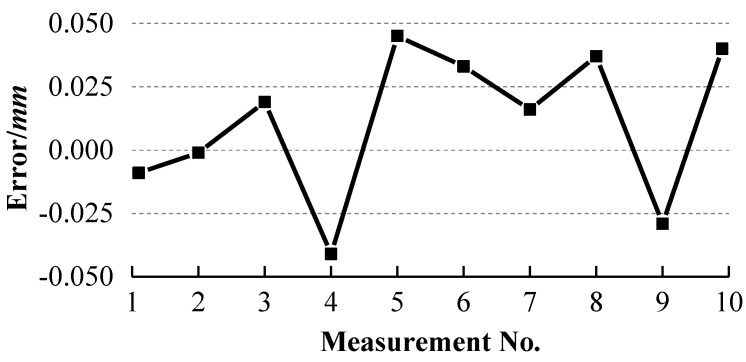
Distance errors between two spheres with our system.

The ball arm was placed at ten different positions with different orientations on the glass plate. It was measured at each position and the collected laser points are used to fit a sphere. The fitted radii of sphere 1 and sphere 2 and the distances between them are listed in Table 1. The errors between the standard radii and the measured radii are calculated and shown in Figure 13. The errors between the standard distance and the measured distances are computed and presented in Figure 14.

**Figure 15:**
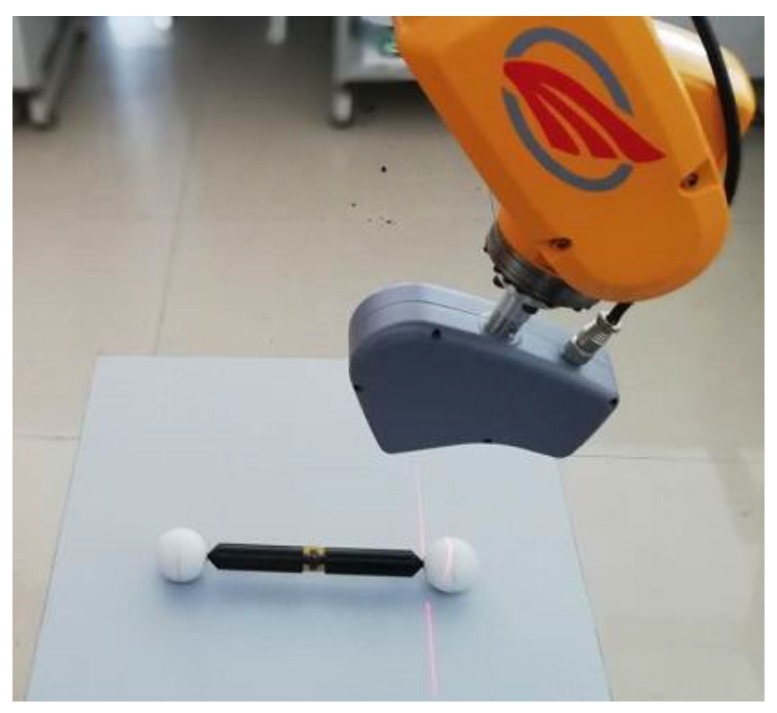
The measurement of ball arm by AASM.

**Figure 16:**
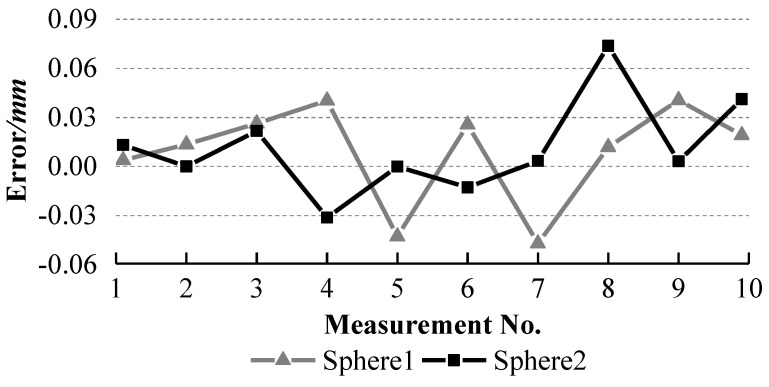
Errors between the standard radii and the measured radii of the two spheres with AAMS.

**Figure 17:**
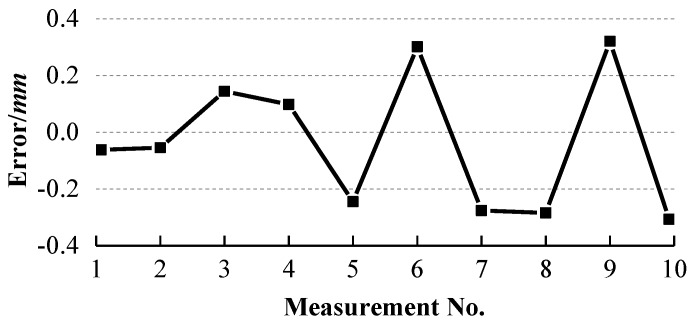
Distance errors between two spheres with AAMS.

To evaluate the accuracy of the system, one AAMS [21] (see Figure 15) is introduced to measure the ball arm ten times at ten different positions and orientations, similar to our system, on one plane of its working rang (700 mm × 500 mm × 400 mm) and the radius errors of two spheres and their distance errors are shown in Figure 16 and Figure 17 respectively.

It can be observed from Figure 13 and Figure 14 that the errors of the two radii and their distance errors are within ±0.04 mm and ±0.05 mm respectively with our system, while Figure 16 and Figure 17 show that the radius errors of two spheres are within ±0.07 mm but their distance errors are fluctuated largely depending on the ball arm’s positions, about ±0.3 mm, with the AAMS. The results indicate the high accuracy of our system.

#### 5.2.2. Random Error Test

**Figure 18:**
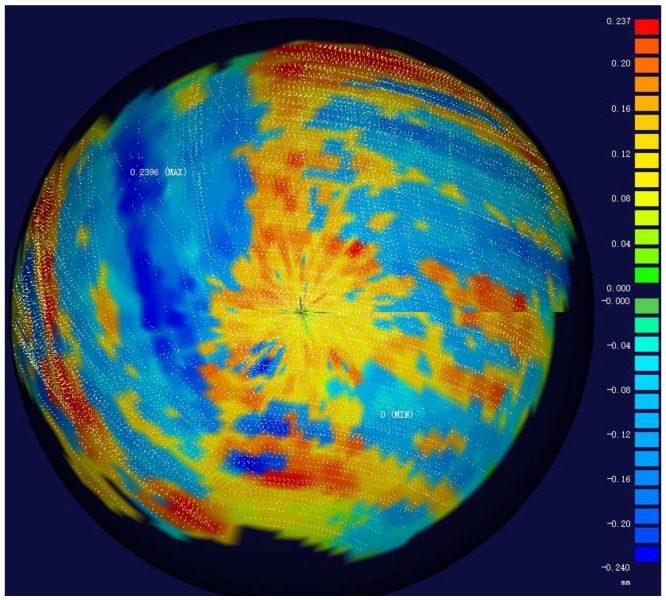
Distribution of the errors from the scatter points to the fitted sphere surface in our system.

To test the random error of our system, the distance errors from the scatter points to the fitted sphere surface are tested. Fitting sphere using the surface points of the sphere1 obtained at Section 5.2.1, the distances distribution from the surface points got by our system to the fitted sphere were obtained, shown in Figure 18. Table 2 and Table 3 show the maximal distance errors from the surface points to the fitted spheres of the ten times with our system and with AAMS respectively.

It can be seen that all the distance errors from the surface points to the fitted sphere are within ±0.25 mm with our system, while the maximum distance of AAMS is only 0.151 mm. The reason is that the scanning path of arm robot can be set, so that it can scan the spheres orderly and get only one layer of laser points. In both systems, the positive errors and the negative errors are distributed approximately symmetrically.

### 5.3. Working Efficiency Test

With the known the marked points stuck on the workpiece measured by TRITOP system, the process of scanning one workpiece in this study is composed by capturing images, extracting the centers of marked points and the centers of laser stripe, matching up the corresponding 3D coordinates of the marked points in $o_{1}x_{1}y_{1}z_{1}$ and $o_{w}x_{w}y_{w}z_{w}$, establishing the transformation from the $o_{1}x_{1}y_{1}z_{1}$ to the $o_{w}x_{w}y_{w}z_{w}$ and transforming the laser stripe into $o_{w}x_{w}y_{w}z_{w}$. The frame rate of the two cameras is 60 frames/s, the shutter time is 8 ms and thus the time of capturing an image is 24.7 ms. A time-consuming test shows the time of extracting the centers of marked points and the laser stripe in both images is about 17.5 ms. Therefore, the total time for obtaining one laser stripe in $o_{w}x_{w}y_{w}z_{w}$ is about 42.2 ms. In other words, about 23 laser lines could be got in one second. The maximum laser point number obtained in per second is 14,720 if the 640 points on a line are all sampled.

### 5.4. Application

**Figure 19:**
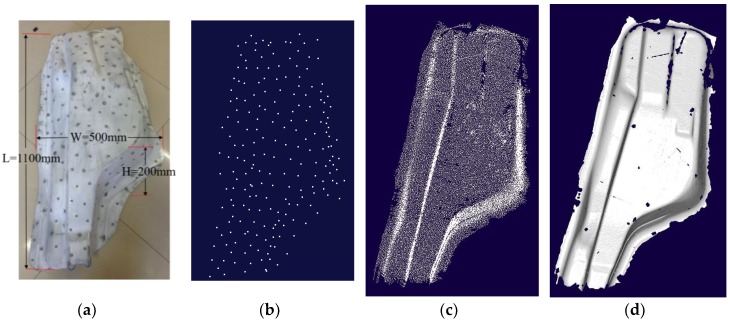
The contour of large-sized workpiece measured by the handheld scanning system in this study. (**a**) the workpiece with typical structures for testing the system performance; (**b**) the measured marked points by TRITOP system; (**c**) the laser points reduced to a third by the system studied; (**d**) the shaped of (**c**) generated in “Imageware.”

**Figure 20:**
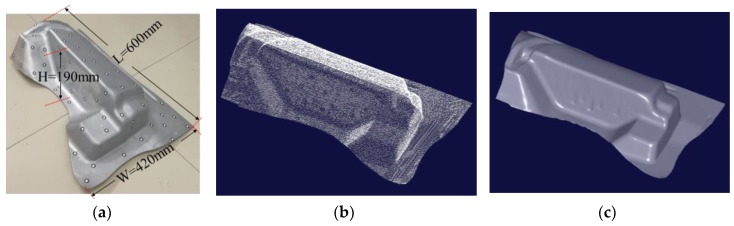
The contour of medium-sized workpiece measured by the handheld scanning system in this study. (**a**) The medium-sized workpiece with typical structures for testing the system performance; (**b**) the measured laser points reduced to a third by the system studied (**c**) the shaped of (**b**) generated in “Imageware.”

Two workpieces shown in Figure 19a and Figure 20a with the size 1100 mm × 500 mm × 200 mm and 600 mm × 420 mm × 190 mm respectively are measured to test the performance of this system.

For the workpiece in Figure 19, to keep at least five points are obtained in $o_{1}x_{1}y_{1}z_{1}$, 221 marked points are randomly stuck on the workpiece, shown in Figure 19. Considering sticking one marked point on the workpiece spending about 1 s, the time consumption of this part is within 4 min. Their 3D coordinates in $o_{w}x_{w}y_{w}z_{w}$ are measured by TRITOP system, which takes about 5 min, shown in Figure 19b. In order to guarantee high accuracy, only more than five marked points are matched up in $o_{1}x_{1}y_{1}z_{1}$ and $o_{w}x_{w}y_{w}z_{w}$, could the obtained laser points in $o_{1}x_{1}y_{1}z_{1}$ be transformed into $o_{w}x_{w}y_{w}z_{w}$. About 15 min is spent to scan this workpiece, with the number of the collected laser points, 4,284,509. Figure 19c presents the reduced laser points by the rule of sampling one point from three points evenly. Figure 19d depicts the shaped form of Figure 19c by the software “imageware.”

For the workpiece in Figure 20a, 86 marked points are randomly stuck on the workpiece. The contour of this workpiece measured by this scanning system is shown in Figure 20b,c. The time expenditure to scan this workpiece is about 12 min to get as much information as we can, especially the edge area and at the regions with large curvature.

It can be seen from Figure 19b,c that the TRITOP system can only get the marked points, while the scanning system in this study can obtain the laser points covering the whole workpiece. From the Figure 19c,d, we can also find that there is no laser point near the edge of the workpiece and at the regions with large curvature. To get the laser points in the edge area and at the regions with large curvature of workpiece in Figure 20b, more time is spent. It is influenced by the number of marked points, the light noises, the posture of scanning system and so forth. Especially, the number of the marked points captured by two cameras simultaneously usually is less than that captured in the other regions and the number of the marked points matched up correctly is difficult to get five.

### 5.5. Discussion

The proposed handheld 3D laser scanning system can obtain the whole contours of typical large-sized workpieces with many features on site with acceptable accuracy and time expenditure. The system’s valid depth of field is $280\;\mathrm{mm}\leq L_{2}\leq 350\;\mathrm{mm}$, the valid view field is about 300 mm×300 mm. To get the contours, it needs the TRITOP system to measure the marked points stuck on the whole workpiece. To get an acceptable accuracy, usually 8~10 marked points (at least 5 points) should be captured synchronously by both cameras in the common view field, 300 mm×300 mm.

The accuracy of this system is tested by evaluating the radii of spheres and their distances, with errors within ±0.05 mm. The cloud thickness is mainly within ±0.25 mm. The errors are distributed evenly based on the marked points measured by TRITOP system, without accumulated errors. The accuracy is relevant to the coordinates of marked points measured by TRITOP system, the internal and external parameters of the scanning system and the transformation relationship between $o_{1}x_{1}y_{1}z_{1}$ and $o_{w}x_{w}y_{w}z_{w}$ in each moment.

The performance of the system is verified by scanning a large-sized workpiece (1100 mm × 500 mm × 200 mm) and a medium-sized workpiece (600 mm × 420 mm × 190 mm) with complex features. The time consumption includes three parts: the time of sticking the marked points on the workpiece, the time of measuring the coordinates of marked points by TRITOP system and the time of scanning the contour with this system, which is relevant to the size of workpiece. The contours of workpieces in Figure 19 and Figure 20 can be reconstructed in 25 min and 20 min respectively.

But there are also some defects to be improved. The edge of the workpiece and the regions with large curvature are difficult to be obtained. The main reason is that the marked points in $o_{1}x_{1}y_{1}z_{1}$ got by the binocular stereo vision system in these regions are difficult to be detected, which leads to the failure of transformation between $o_{1}x_{1}y_{1}z_{1}$ and $o_{w}x_{w}y_{w}z_{w}$.

## 6. Conclusions

This paper presents a mobile 3D scanning system based on the known marked points obtained by the TRITOP system technique beforehand. Compared with the existed methods, (1) it can measure the 3D contour of large-sized workpieces on site with complex features by overcoming some problems in current 3D scanning methods, such as range limitation and sheltering; (2) the system is easy to be used with low demand to the operators, the scanning process can be stopped and discontinuous to check and get laser points; (3) its errors are distributed evenly.

The accuracy of the system is tested by measuring a ball arm with two standard spheres. The ball arm is placed on a glass plane, on which many marked points are randomly stuck and measured by a TRITOP system. The distance errors between the two sphere centers are within ±0.05 mm, the radius errors of two spheres are all within ±0.04 mm and the distance errors from the surface points to the fitted sphere are within ±0.25 mm. Experimental results demonstrate that the system enjoys high accuracy and high stability and can satisfy the accuracy demand of measuring the 3D contours of large-sized workpieces on site.

The measuring results of two workpieces with complex structure also indicate the difficulty in collecting data points near the edge of the workpiece and at the regions with large curvature. Because the number of the marked points correctly matched in $o_{1}x_{1}y_{1}z_{1}$ and $o_{w}x_{w}y_{w}z_{w}$ in these regions is less than five. To increase the matched number, it is necessary to increase the density of the marked points on the object or enlarge the working range of the system.

## Figures and Tables

**Table 1:** The fitted radii of the two spheres and the distances between them.

Measurement No.	Radius1 (mm)	Radius2 (mm)	Distance (mm)
1	20.037	20.069	198.504
2	20.016	20.034	198.512
3	20.043	20.036	198.532
4	19.995	20.076	198.472
5	20.039	20.020	198.558
6	20.054	20.097	198.546
7	20.018	20.053	198.529
8	19.996	20.032	198.550
9	20.042	20.081	198.481
10	20.064	20.094	198.553
Average	20.0304	20.0592	198.523

**Table 2:** Maximum distance errors from the scatter points to the fitted sphere surface with our system.

Measurement No.	1	2	3	4	5	6	7	8	9	10
Max distance1 ^1^/mm	0.235	0.222	0.216	0.245	0.239	0.242	0.229	0.219	0.233	0.240
Max distance2 ^2^/mm	0.252	0.224	0.254	0.200	0.240	0.232	0.253	0.223	0.241	0.235

^1^ Max distance1 is the maximum distance from the scatter points outside the sphere to the fitted sphere surface. ^2^ Max distance2 is the maximum distance from the scatter points inside the sphere to the fitted sphere surface.

**Table 3:** Maximum distance errors from the scatter points to the fitted sphere surface with AAMS.

Measurement No.	1	2	3	4	5	6	7	8	9	10
Max distance1/mm	0.081	0.095	0.107	0.089	0.139	0.127	0.151	0.090	0.142	0.149
Max distance2/mm	0.117	0.097	0.119	0.143	0.092	0.105	0.126	0.134	0.095	0.113

## References

[1] Amans O.C., Beiping W., Ziggah Y.Y., Daniel A.O. (2013). The need for 3D laser scanning documentation for select Nigeria cultural heritage sites. Eur. Sci. J..

[2] Price G.J., Parkhurst J.M., Sharrock P.J., Moore C.J. (2012). Real-time optical measurement of the dynamic body surface for use in guided radiotherapy. Phys. Med. Biol..

[3] Stevanovic N., Markovic V.M., Nikezic D. (2017). New method for determination of diffraction light pattern of the arbitrary surface. Opt. Laser Technol..

[4] Ke F., Xie J., Chen Y., Zhang D., Chen B. (2014). A fast and accurate calibration method for the structured light system based on trapezoidal phase-shifting pattern. Optik.

[5] Suresh V., Holton J., Li B. (2018). Structured light system calibration with unidirectional fringe patterns. Opt. Laser Eng..

[6] Cuesta E., Suarez-Mendez J.M., Martinez-Pellitero S., Barreiro J., Zapico P. (2017). Metrological evaluation of Structured Light 3D scanning system with an optical feature-based gauge. Procedia Manuf..

[7] Ganganath N., Leung H. Mobile robot localization using odometry and kinect sensor. Proceedings of the IEEE Conference Emerging Signal Processing Applications.

[8] Tang Y., Yao J., Zhou Y., Sun C., Yang P., Miao H., Chen J. (2018). Calibration of an arbitrarily arranged projection moiré system for 3D shape measurement. Opt. Laser Eng..

[9] Zhong M., Chen W., Su X., Zheng Y., Shen Q. (2013). Optical 3D shape measurement profilometry based on 2D S-Transform filtering method. Opt. Commun..

[10] Zhang Z., Jing Z., Wang Z., Kuang D. (2012). Comparison of Fourier transform, windowed Fourier transform, and wavelet transform methods for phase calculation at discontinuities in fringe projection profilometry. Opt. Lasers Eng..

[11] Bleier M., Nüchter A. (2017). Towards robust self-calibration for handheld 3D line laser scanning. Int. Arch. Photogramm. Remote Sens. Spat. Inf. Sci..

[12] Zhang S. (2013). Handbook of 3D Machine Vision: Optical Metrology and Imaging.

[13] Koutecký T., Paloušek D., Brandejs J. (2013). Method of photogrammetric measurement automation using TRITOP system and industrial robot. Optik Int. J. Light Electron Opt..

[14] Gmurczyk G., Reymer P., Kurdelski M. (2011). Global FEM Model of combat helicopter. J. KONES Powertrain Transp..

[15] Xu H., Ren N. (2006). Working Principle and System Calibration of ATOS Optical Scanner. Tool Eng..

[16] Xie Z., Lu W., Wang X., Liu J. (2015). College of Engineering, Ocean University of China. Analysis of Pose Selection on Binocular Stereo Calibration. Chin. J. Lasers.

[17] Chen S., Xia R., Zhao J., Chen Y., Hu M. (2017). A hybrid method for ellipse detection in industrial images. Pattern Recognit..

[18] Sun Q., Liu R., Yu F. (2016). An extraction method of laser stripe centre based on Legendre moment. Optik.

[19] Tian Q., Zhang X., Ma Q., Ge B. (2016). Utilizing polygon segmentation technique to extract and optimize light stripe centerline in line-structured laser 3D scanner. Pattern Recognit..

[20] Sun Q., Chen J., Li C. (2015). A robust method to extract a laser stripe centre based on grey level moment. Opt. Laser Eng..

[21] Mu N., Wang K., Xie Z., Ren P. (2017). Calibration of a flexible measurement system based on industrial articulated robot and structured light sensor. Opt. Eng..

